# Carrier frequencies of eleven mutations in eight genes associated with primary ciliary dyskinesia in the Ashkenazi Jewish population

**DOI:** 10.1002/mgg3.124

**Published:** 2014-12-06

**Authors:** Anastasia M Fedick, Chaim Jalas, Nathan R Treff, Michael R Knowles, Maimoona A Zariwala

**Affiliations:** 1 Department of Microbiology and Molecular Genetics, Rutgers-Robert Wood Johnson Medical SchoolPiscataway, New Jersey; 2 Reproductive Medicine Associates of New JerseyBasking Ridge, New Jersey; 3 Bonei Olam, Center for Rare Jewish Genetic DisordersBrooklyn, New Jersey; 4 Department of Medicine, University of North Carolina School of MedicineChapel Hill, North Carolina; 5 Department of Pathology/Lab Medicine, University of North Carolina School of MedicineChapel Hill, North Carolina

**Keywords:** Ashkenazi, carrier frequency, Kartagener syndrome, primary ciliary dyskinesia, situs inversus

## Abstract

Primary ciliary dyskinesia (PCD) is a genetically heterogeneous, autosomal recessive disorder that results from functional and ultrastructural abnormalities of motile cilia. Patients with PCD have diverse clinical phenotypes that include chronic upper and lower respiratory tract infections, *situs inversus*, heterotaxy with or without congenital heart disease, and male infertility, among others. In this report, the carrier frequencies for eleven mutations in eight PCD-associated genes (*DNAI1, DNAI2, DNAH5, DNAH11, CCDC114, CCDC40, CCDC65,* and *C21orf59*) that had been found in individuals of Ashkenazi Jewish descent were investigated in order to advise on including them in existing clinical mutation panels for this population. Results showed relatively high carrier frequencies for the *DNAH5* c.7502G>C mutation (0.58%), the *DNAI2* c.1304G>A mutation (0.50%), and the *C21orf59* c.735C>G mutation (0.48%), as well as lower frequencies for mutations in *DNAI1*, *CCDC65*, *CCDC114*, and *DNAH11* (0.10–0.29%). These results suggest that several of these genes should be considered for inclusion in carrier screening panels in the Ashkenazi Jewish population.

## Introduction

Primary ciliary dyskinesia (PCD, MIM: 244400) is a genetically heterogeneous, autosomal recessive disorder that results from functional and ultrastructural abnormalities of motile cilia. Phenotypes of PCD can be diverse, with irregularities in motile respiratory cilia causing chronic upper and lower respiratory tract infections, in embryonic nodal cilia causing *situs inversus* and heterotaxy with or without congenital heart disease, and in spermatozoa flagella causing male infertility, among others clinical features (Zariwala et al. 2007; Escudier et al. 2009; Leigh et al. 2009; Knowles et al. 2013a). PCD can be diagnosed in various manners including the detection of ultrastructural defects by transmission electron microscopy, abnormal ciliary beat frequencies or patterns, low levels of nasal nitric oxide, the presentation of typical clinical features, and mutation analysis in ciliary genes (Zariwala et al. 2007; Escudier et al. 2009; Leigh et al. 2009, 2013; Knowles et al. 2013a; Svobodova et al. 2013).

Primary ciliary dyskinesia has extensive locus and allelic heterogeneity that makes it challenging for genetic diagnoses. Due to the high number of causative PCD mutations, tiered mutation screening methods have been suggested for PCD patients (Hornef et al. 2006; Zariwala et al. 2006; Djakow et al. 2012). In tiered screenings, mutations are ranked based on their prevalence in the PCD population, with the most common mutations screened first in an effort to increase efficiency. Founder effects have resulted in certain recessive mutations to appear more frequently in specific ethnic populations as well, which allows for a different ranking system to be made based on the frequency of mutations in a specific ethnic group or geographic isolates. An example of such effects has been seen in PCD where a splice-site mutation in *RSPH4A* (MIM 612649, 612647) is found in people of Hispanic origin from Puerto Rico (Daniels et al. 2013). Since the Ashkenazi Jewish population has an increased prevalence of recessive mutations due to past founder effects (Bray et al. 2010), carrier frequencies were performed for eleven mutations in eight PCD genes that had been observed in affected individuals of Ashkenazi Jewish descent in order to advise on screening for them.

Of the 11 mutations evaluated, six were in four genes known to cause defective ciliary outer dynein arm (ODA) complexes (Hornef et al. 2006; Zariwala et al. 2006; Knowles et al. 2013b). These included mutations in *DNAI1* (OMIM# 604366) (NM_012144.2) c.1490G>A (r.1402_1569del) (p.Arg468_Lys523del) (Zariwala et al. 2006), *DNAI2* (OMIM# 605483) (NM_023036.4) c.1304G>A (p.Trp435*) (Knowles et al. 2013b), *CCDC114* (OMIM# 615038) (NM_144557.3) c.939delT (p.His313Glnfs*14) (Knowles et al. 2013b), and three in *DNAH5* (OMIM# 603335) (NM_001369.2): c.7502G>C (p.Arg2501Pro) (Hornef et al. 2006), novel c.5545G>A (p.Ala1849Thr) and novel c.6988+2T>C (g.IVS42+2T>C) (p. splice?). Two of the remaining mutations were in *CCDC40* (OMIM# 613799) (NM_017950.3) c.248delC (p.Ala83Valfs*84) (Becker-Heck et al. 2011; Antony et al. 2012) which causes defective inner dynein arm (IDA) complexes together with microtubular disorganization in a subset of cilia, and *C21orf59* (OMIM# 615494) (NM_021254.2) c.735C>G (p.Tyr245*) (Austin-Tse et al. 2013) which causes the absence of both ODAs and IDAs. The remaining three mutations were from two genes that do not cause any detectable ciliary ultrastructural defects (Schwabe et al. 2008; Knowles et al. 2012; Austin-Tse et al. 2013; Horani et al. 2013) and included the c.877_878delAT (p.Ile293Profs*2) in *CCDC65* (OMIM# 611088) (NM_033124.4) (Austin-Tse et al. 2013; Horani et al. 2013) and two mutations in *DNAH11*(OMIM# 603339) (NM_001277115.1): c.6244C>T (p.Arg2082*) and c.11929G>T (p.Glu3977*) (Knowles et al. 2012).

## Materials and Methods

### Ethics statement

The samples used in this study were obtained with written patient consent from self-identified Ashkenazi Jews enrolled in the carrier testing Dor Yeshorim program (Ekstein and Katzenstein 2001) to be used for research purposes. Consent form information included that patient material would be used for clinical testing and that excess material would be de-identified and used for research purposes to characterize single-gene disorders in the Ashkenazi Jewish population. The positive control samples came from the Institutional review board approved research cohort for the protection of the rights of human subjects that were recruited under the auspices of the Genetic Disorders of Mucociliary Clearance consortium. Institutional review board permission was not required for the control samples used in the carrier frequency study because all sample identifiers were removed prior to receipt by the laboratory where the TaqMan assays were carried out (45 CFR part 46.101(b)(4)).

### Patients

The positive control samples came from the individuals harboring mutations who self-identified as being from an Ashkenazi Jewish descent. The genotypes for the majority of the positive controls for the mutations in *DNAH11* (NM_001277115.1), *DNAI1* (NM_012144.2), *DNAI2* (NM_023036.4), *CCDC114* (NM_144577.3), *CCDC40* (NM_017950.3), *C21orf59* (NM_021254.2), and *CCDC65* (NM_033124.4) have previously been published (Zariwala et al. 2006; Antony et al. 2012; Knowles et al. 2012; Austin-Tse et al. 2013; Knowles et al. 2013b). In addition, for the previously known c.7502G>C missense mutation (Hornef et al. 2006)and a novel c.6988+2T>C splice-site mutation in *DNAH5* (NM_001369.2), we used gDNA from the affected individual (#826), who harbored both mutations. Individual #826 had an affected sibling with the identical *DNAH5* genotypes and both unaffected parents were carriers; thus, indicating both mutations in the affected individuals were inherited in *trans*. For the c.5545G>A mutation in *DNAH5*, the gDNA from an unaffected carrier mother of Ashkenazi descent was used. This mutation was observed in a PCD affected individual who harbored a splice-site mutation on the *trans* allele that was inherited from a non-Ashkenazi unaffected father; thus the splice-site mutation was not evaluated.

### Assay design and validation

To design the genotyping assays, the full sequence of each gene was obtained from the National Center for Biotechnology Information (NCBI) (U.S. National Library of Medicine, Bethesda, MD). Roughly 200 base pairs upstream and downstream of the mutation site were selected and repetitive sequences and SNPs were masked using Repeat Masker (Institute for Systems Biology, Seattle, WA) and NCBI specialized Basic Local Alignment Search Tool (BLAST) using the SNP Flanks option, respectively. The assays were then made in File Builder software (Life Technologies [LTI], Carlsbad, CA) with sequence-specific forward and reverse primers to amplify the polymorphic sequences, and VIC and FAM fluorescent-labeled probes to detect the normal and mutant alleles, respectively (Table1). A no template control consisting of water, three wild-type samples, and one known heterozygous carrier sample were used to validate all of the assays except for the *CCDC40* assay which was validated on homozygous affected gDNA. The genotypes for all of the control samples were confirmed using Sanger Sequencing. The samples and assays were plated in duplicate in a 384 well plate along with TaqMan Genotyping Master Mix (LTI) (final volume 5 *μ*L) and run in duplex real-time PCR reactions followed by allelic discrimination on the ABI PRISM® 7900 HT Sequence Detection System using SDS 2.3 software (LTI).

**Table 1:** TaqMan assay design including primer and probe sequences

Genes	Mutations	Forward primer	Reverse primer	VIC probe	FAM probe
*DNAI1*	c.1490G>A	CCTCTTGGAACTGGGCTAAGC	CATGTAGTCAATCTCTTTGTGGAAGTCA	TCTTTTCCCAAGGTTGTG	TTTTCCCAAGATTGTG
*DNAI2*	c.1304G>A	CGTTTTCTTTACCACCAGGATGGA	GGATCGCACTGCTCGAACAT	CTGGATATCTGGGACTTC	CCTGGATATCTAGGACTTC
*DNAH11*	c.6244C>T	GTGCTATTAAGTCTGTCTTGGTTGTG	TCATATTAGCATTGCAGTACCTGATCTTC	ATTTTTATCTCCTCGTTTCAG	TTTTTATCTCCTCATTTCAG
*DNAH11*	c.11929G>T	CAGGAGACGGTGGCAGAAG	CCCAGTGTCCTCCTTTGGAA	TGGCCCTGGAGAAAG	TGGCCCTGTAGAAAG
*DNAH5*	c.6988+2T>C	GGGATATTTTCTACGCTTTGGAGGAA	GTATAGCCTCCAAGGATTCTATCTAGAAGT	TTGTAATTTATCTTCTACCTTTCT	AATTTATCTTCTGCCTTTCT
*DNAH5*	c.7502G>C	TCGCGCTGCTGTGGAG	CGCAGCCAGAGCTCCAG	ACGGACGGCGCCGC	ACGGACCGCGCCGC
*DNAH5*	c.5545G>A	TGATATGGACACGGGATTCAGAAGA	CTCCAGGAAAGCCTGATTAGTTTTC	AGCCCTTAGAAATGCCAAGT	AGCCCTTAGAAATACCAAGT
*CCDC40*	c.248delC	AAGCGGAAGCTGCAATTGA	TCCTCTTCGCTTTCAGCATCTC	AGGAGGCTGTGTCCTA	AGGAGGTGTGTCCTATG
*CCDC65*	c.877_878delAT	GCCATAACTATTTCAAAAGGCAAGATCA	CGCAGTTGTACAAGGACCAATTC	ATGAGAACCGGTATATCCGTA	ATGAGAACCGGTATCCGTA
*CCDC114*	c.939delT	TCATCAACGAGCAGAACTTGGA	AGGCCTCACTCACCTCCTTGA	CTGGAGCATGTGCAGGA	AGCAGTGCAGGAAGA
*C21orf59*	c.735C>G	GAGGAGCAGAAGCAGCTGAT	GCACTGCAGAAAGCCCATCT	TGTCTTCTGTGATAGTACAGC	TGTCTTCTGTGATACTACAGC

GenBank reference sequence and version number for the genes studied: *DNAI1* (NM_012144.2), *DNAI2* (NM_023036.4), *DNAH11* (NM_001277115.1), *DNAH5* (NM_001369.2), *CCDC40* (NM_017950.3), *CCDC65* (NM_033124.4), *CCDC114* (NM_144577.3), and *C21orf59* (NM_021254.2).

### Carrier frequency study

For the carrier frequency study, ∽1000 samples were analyzed for each mutation. The gDNA samples were not normalized prior to plating, but almost all samples fell within the suggested range of 1–20 ng (LTI). The plates were run on the GeneAmp® PCR System 9700 (LTI) at the following setting: holds at 50°C for 2 min and 95°C for 10 min, and then 40 cycles at 95°C for 15 sec and 60°C for 1 min. Allelic discrimination was then performed on the ABI PRISM® 7900 HT Sequence Detection System using SDS 2.3 software and the data were analyzed using TaqMan Genotyper v1.1 software (LTI). Any samples that did not amplify were not included in the carrier frequency calculations. The samples from the initial validation were used as controls. The Wilson score interval (Wilson 1927) was used to calculate the confidence intervals (CI) for carrier frequencies.

## Results

**Figure 1:**
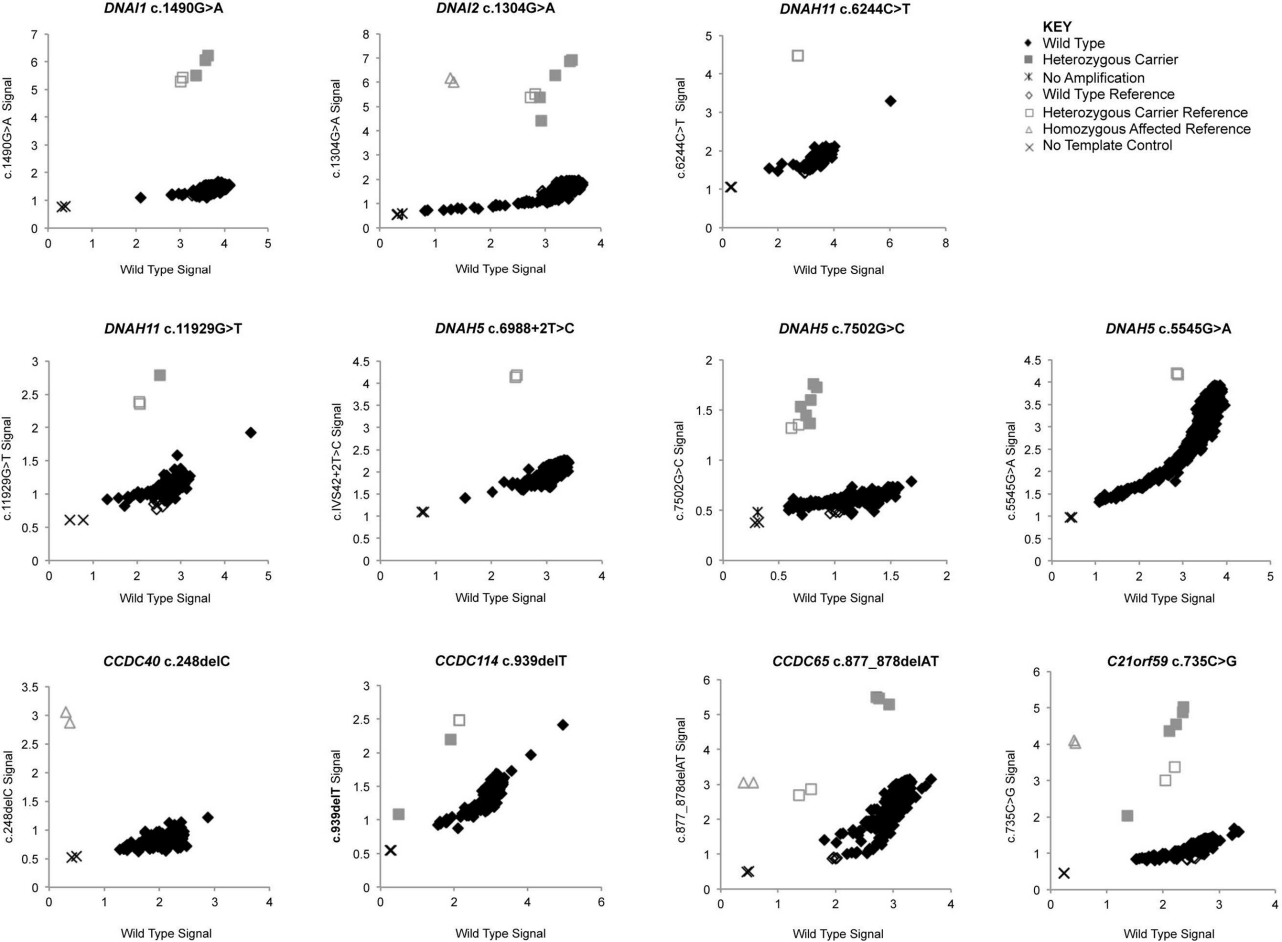
Carrier frequency allelic discrimination plots. For all plots, the VIC probe (wild-type allele) is represented by the *X*-axis, and the FAM probe (mutant allele) is represented by the *Y*-axis. Sterile water was used as the no template control. The GenBank reference sequence and version number for the genes studied are as follows: *DNAI1* (NM_012144.2), *DNAI2* (NM_023036.4), *DNAH11* (NM_001277115.1), *DNAH5* (NM_001369.2), *CCDC40* (NM_017950.3), *CCDC65* (NM_033124.4), *CCDC114* (NM_144577.3), and *C21orf59* (NM_021254.2).

The initial validation of the assays on a small scale yielded 100% genotyping accuracy. The carrier frequency experiments were then performed on ∽1000 samples from individuals of Ashkenazi Jewish descent. The carrier frequency results were as follows: 0.28% (CI 0.09–0.83%) for the *DNAI1* c.1490G>A mutation, 0.50% (CI 0.21–1.16%) for the *DNAI2* c.1304G>A mutation, 0.10% (CI 0.02–0.54%) for the *DNAH11* c.11929G>T mutation, 0.58% (CI 0.27–1.26%) for the *DNAH5* c.7502G>C mutation, 0.19% (CI 0.05–0.69%) for the *CCDC114* c.939delT mutation, 0.29% (CI 0.10–0.85%) for the *CCDC65* c.877_878delAT mutation, and 0.48% (CI 0.20–1.12%) for the *C21orf59* c.735C>G mutation. No carriers were detected for the *DNAH5* c.6988+2T>C and c.5545G>A, the *DNAH11* c.6244C>T, or the *CCDC40* c.248delC mutations. TaqMan allelic discrimination result plots can be seen in Figure1 for all of the mutations, and a summary of the carrier frequencies can be seen in Table2. Samples identified as being heterozygous carriers were confirmed by Sanger Sequencing (if gDNA was available), and all genotypes were concordant between the two methods.

**Table 2:** Carrier frequency of 11 mutations in eight primary ciliary dyskinesia-associated genes

Genes	Mutations	No. of individuals wild type	No. of. individuals heterozygous carrier^1^	Carrier frequency (%) and confidence interval (%)
*DNAI1*	c.1490G>A	1052	3	0.28 (0.09–0.83)
*DNAI2*	c.1304G>A	1000	5	0.50 (0.21–1.16)
*DNAH11*	c.6244C>T	1052	0	0.00
*DNAH11*	c.11929G>T	1051	1	0.10 (0.02–0.54)
*DNAH5*	c.6988+2T>C	1050	0	0.00
*DNAH5*	c.7502G>C	1036	6	0.58 (0.27–1.26)
*DNAH5*	c.5545G>A	1051	0	0.00
*CCDC40*	c.248delC	1052	0	0.00
*CCDC65*	c.877_878delAT	1032	3	0.29 (0.10–0.85)
*CCDC114*	c.939delT	1054	2	0.19 (0.05–0.69)
*C21orf59*	c.735C>G	1031	5	0.48 (0.20–1.12)

1 None of the individuals were identified as being homozygous for the mutations.

## Discussion

Because of the extensive heterogeneous nature of PCD, knowing if certain mutations are more prevalent in a specific ethnic population can help prioritize variants for expeditious screening. In this study, 11 different mutations represented by eight genes that had all been previously found in individuals of Ashkenazi Jewish descent were examined. Of these, seven mutations representing seven genes were found to have carrier frequencies ranging from 0.1% to 0.58% in the population.

Of all the mutations screened, the highest frequency (0.58%) was found for the c.7502G>C mutation in the *DNAH5* gene. Mutations in this gene are the most common in PCD patients in the general population (Hornef et al. 2006; Failly et al. 2009), accounting for 28% of all PCD families and 49% of PCD families that have ODA defects (Zariwala et al. 2007). The 0.48% frequency of the c.735C>G mutation in *C21orf59* and the 0.29% for the c.877_878delAT mutation in *CCDC65* are believed to be founder mutations, which explains their relatively high frequencies (Austin-Tse et al. 2013). The 0.29% carrier frequency for the c.877_878delAT *CCDC65* was slightly lower than the 0.41% previously detected in this population (Horani et al. 2013), which may reflect that this study was conducted on the orthodox as opposed to the general Ashkenazi population. Other mutations that had been previously reported to be at mutation hotspots for PCD patients had no carriers detected, such as the c.248delC mutation in *CCDC40* (Antony et al. 2012).

Based on the various frequencies found, we suggest that the seven mutations that had carriers detected be recommended for inclusion in mutation screening panels specific for the Ashkenazi population since there is a chance of compound heterozygosity based on the prevalence of the various mutations. While none of the mutations meet the inclusion criteria of a 1% frequency set by the American College of Medical Genetics (ACMG) (Gross et al. 2008) the advances made in high-throughput screening programs (Fedick et al. 2013) have made screening for additional and/or rare mutations possible and affordable, with many commercial laboratories already offering ethnic screening panels that extend beyond the scope of mutations specifically recommended by the ACMG and other such organizations (Lazarin et al. 2013) Since the ACMG, along with the American College of Obstetricians and Gynecologists, has acknowledged that individuals may want to be screened for additional disorders, we suggest that information for all of the PCD genes and mutations studied here be made available when receiving genetic counseling to aid patients in making informed decisions (Monaghan et al. 2008; ACOG Committee on Genetics 2009).
